# HPV transcription in skin tumors

**DOI:** 10.1371/journal.pone.0217942

**Published:** 2019-05-31

**Authors:** Emilie Hultin, Laila Sara Arroyo Mühr, Camilla Lagheden, Joakim Dillner

**Affiliations:** 1 Division of Pathology, Department of Laboratory Medicine, Karolinska Institutet, Stockholm, Sweden; 2 Clinical Pathology/Cytology, Karolinska University Laboratory, Karolinska University Hospital, Stockholm, Sweden; Istituto Nazionale Tumori IRCCS Fondazione Pascale, ITALY

## Abstract

**Background:**

Although more than 95% of viral sequences found in skin tumors typically belong to human papillomaviruses (HPVs), HPV transcription has so far not been detected. As current technology allows very deep transcriptome sequencing, we examined skin tumors and precursor lesions for HPV transcription.

**Method:**

Fresh frozen biopsies from 12 skin specimens (11/12 were positive for HPV DNA) were subjected to total RNA sequencing. The cervical cancer cell line CaSki was included as a positive control for HPV transcription.

**Results:**

HPV RNA was detected and confirmed in 1/12 skin lesions at a median depth of 66 million reads per sample. One specimen was positive for HPV 110 transcripts mapping to E6, E7, E2/E4 and L2 open reading frames, as well as to a spliced E1^E4 transcript.

**Conclusion:**

In conclusion, the study revealed that a minority of skin lesions contains HPV transcripts and that HPV DNA detection does not predict HPV transcriptional activity.

## Introduction

Human papillomaviruses (HPVs) infect keratinocytes in both mucosa and skin and can cause several diseases from benign lesions to malignant tumors [1, 2]. Cutaneous HPV types are often found both in healthy skin as well as in skin lesions such as benign skin warts [3], non-melanoma skin cancers (NMSCs) including squamous cell carcinoma (SCC) and basal cell carcinoma [4], actinic keratosis (precancerous skin lesions), keratoacanthomas (benign skin tumors) and Bowen’s disease (SCC in situ). The increased incidence of NMSCs among immunosuppressed patients has suggested that infectious agent(s) may be involved [5–9]. Metagenomic sequencing has revealed that more than 95% of the viral sequences present in skin tumor samples belong to the family Papillomaviridae [10], mostly to the cutaneous types belonging to beta and gamma genera [11, 12]. Patients with the skin disorder epidermodysplasia verruciformis, where beta-human papillomavirus infection is a phenotypic condition, develop NMSCs and are suspected to have a defect of immunity to beta-HPV [13]. This suggests that HPV may be involved in NMSC development.

The traditional methods to identify HPV-related lesions/cancer are based on PCR, targeting usually the *L1* gene which is the most conserved gene within the HPV genome [14]. PCR methods are biased to detect mostly known HPV types that bind specifically to designed primers and probes [15, 16]. Unknown HPV types may diverge from the primers/probe sequences in their nucleotide sequences and may escape amplification [17]. Most of the authors detected a broad spectrum of HPV types in skin tumors [4, 18, 19]. Among them, HPV type 197 appears to be particularly common [20].

Detection of HPV DNA does not necessarily mean that an HPV infection has been detected, as it may represent a viral deposition on the skin surface [21]. Simple pre-treatment of the skin surface before sampling significantly reduces the proportion of HPV positive samples (12). Many studies report that HPV is detected in very low amounts in skin tumors (<1 copy/cell) [22–24], but there are occasional reports of skin tumors that do contain high or very high amounts of HPV [15, 16].

Metagenomic sequencing without prior PCR allows detection of all HPVs present in a sample, without previous knowledge on which types might be present [17, 18, 25]. Although metagenomics studies of viral DNA have found a lot of HPVs in skin tumor samples [10, 12, 18], whole transcriptome studies have so far not detected any HPV transcription in these tumors [26, 27]. Investigation of viral transcription is important, as transcription of viral genes is necessary for viral pathogenicity, as demonstrated in head and neck cancers [28, 29]. The theory of involvement of infections in NMSC after immunosuppression implies expression of an infection-encoded antigen and data on whether there is viral transcription or not is therefore important. As the deep sequencing technology is continuously advancing, we wished to detect active HPV infection analyzing the whole transcriptome in skin tumors and pre-malignant lesions using RNA sequencing.

## Materials and methods

### Sample material and RNA extraction

Written informed consent was obtained from all participants in the clinical study. The study was approved by the Ethical review Committee of Karolinska Institutet and of Lund University (Sweden) (02–135, LU556-02). We included a set of 12 fresh frozen biopsies from immunocompetent patients (1 SCC, 2 Bowen´s diseases, 7 actinic keratosis and 2 keratoacanthomas) prepared for RNA analysis. Samples had been whole genome sequenced using MiSeq or HiSeq Illumina platforms in a previous study [20]. The RNA integrity (RIN) of the samples were checked using Bioanalyzer (Agilent Technologies, US), where the algorithm assigns a 1 to 10 RIN score, where score 10 RNA is completely intact [30]. The sample with the highest RIN score for each of the two hospitals Lund University Hospital and Sahlgrenska University Hospital (LH116 with RIN score 9.2 and Sahl77 with RIN score 9.5) as well as a set of HPV DNA-positive samples were included for the RNA-sequencing. Two-millimeter biopsies were taken from the skin lesion (after tape stripping to remove possible environmental deposition of viruses). The biopsies were divided in two equal parts, one for DNA and one for RNA analysis. The RNA-part was treated with RNAlater and kept at -70°C.

RNA was extracted using RNeasy Lipid Tissue Mini kit (Qiagen), following the manufacturer´s instructions, including the DNase digestion, and kept at -70°C until further analysis.

A cervical cancer cell line (CaSki (ATCC CRM-CRL-1550)) known to contain approximately 600 copies of HPV type 16 genome integrated into the human genome, was used as a positive control for virus presence and transcription. The cell line was cultured and harvested cells were treated with RNAlater and RNA was extracted using the same RNeasy Lipid Tissue Mini kit (Qiagen), following the manufacturer´s instructions.

### RNA sequencing

The RNA extracted samples (n = 14, 12 skin lesions, 2 CaSki cell line positive controls) together with two negative controls containing lab-grade water, were rRNA depleted, reverse transcribed and ligated to individual indexed adapters using the TruSeq Stranded Total RNA kit according to the user´s guide, Revision E (Illumina, US). All 16 individual libraries were validated, normalized to 2 nM and pooled accordingly (except the negative water controls, which were pooled using the same volume without normalization) before sequencing. Sequencing was performed in the NextSeq500 system (Illumina, US) at 151 paired-end cycles. A total of 4 runs were carried out. The first 2 runs contained a pool of 6 skin lesions, 1 negative control (lab-grade water) and 1 positive control (CaSki cell line) each. In the third run, only two lesions were sequenced. The fourth run was performed as a validation of the HPV 16 RNA-positive specimen in the second run, now sequenced without the CaSki cell line positive control to discard possible index-hopping.

### Bioinformatic analysis

Indices, included in the Illumina adapters, were used to assign raw sequence reads obtained from the NextSeq500 (Illumina) platform to the originating samples. Reads were quality checked and adapter trimmed with Trimmomatic [31]. High quality reads were screened against the human reference genome hg19 using NextGenMap [32] and only reads that did not map to the human genome, with >95% identity over 75% of their length, were considered as non-human and further analyzed for HPV. Reads that did map to the human reference genome were used to assess sample and protocol adequacy by analyzing presence of reads mapping to human reference protein coding genes, actin beta (ACTB) and glyceraldehyde 3-phosphate dehydrogenase (GAPDH).

Once human reads were filtered from the data set, reads from each sample were queried against all HPV protein sequences included in the PaVE database (Papillomavirus Episteme, accessed on 2018-07-28, including all protein sequences from HPV reference and non-reference genomes), using the open source software Diamond blastx with default parameters and—top 1 (to report only alignments whose score is at most 1% lower than the best alignment score for the query) [33]. Samples were considered positive if a minimum of 5 reads were detected for any HPV type.

## Results

### Human reference genes

Specimen adequacy was confirmed for all samples, except for the negative controls, through coverage analysis of human reference genes known to be expressed in skin (ACTB and GAPDH) (S1 Table). All samples (except the negative controls) had high coverage of most of the ACTB and GAPDH exons but not ACTB and GAPDH introns, indicating pure RNA with no or very little DNA content (data showed for the HPV-positive samples LH116 and LH130 for ACTB and GAPDH in S1 Fig and S2 Fig, respectively).

### HPV RNA detection

The Illumina sequencing generated high quality sequencing data with a median of 66 million reads per sample (Table 1). Most of the reads from each specimen (around 78% of total reads) were classified as human. Positive controls for HPV mRNA (CaSki cells) generated more than 89 000 reads for HPV16 transcriptome, and negative controls were negative for HPV. All the aligned, non-human sequences are available at the sequence read archive within the bioproject ID PRJNA493709 (https://www.ncbi.nlm.nih.gov/sra/PRJNA493709).

**Table 1:** Reads obtained with RNA sequencing of skin lesions.

RNA-seq	Specimen	Skin lesion	RIN	Total reads	Human reads (%)	HPV type DNA*	HPV type RNA	HPV RNA reads
Run 1	LH129	KA	7.6	60 872 608	47 423 223 (77.91)	197	Neg	0
	LH130	AK	x	71 330 394	56 091 909 (78.64)	124, 197	16	53
	LH137	AK	7.5	60 301 502	47 878 814 (79.40)	197	Neg	0
	SAHL64	BD	6.7	64 997 620	51 927 719 (79.89)	197	Neg	0
	SAHL73	BD	8.2	77 728 410	61 563 831 (79.20)	197	Neg	0
	SAHL83	SCC	8.0	81 274 014	64 857 251 (79.80)	197	Neg	0
	CASKI	-	x	79 047 250	64 857 251 (71.52)	16	16	132 558
	WATER	-	x	3 842 878	126 505 (3.29)	-	Neg	0
Run 2	LH116	AK	9.2	62 531 732	51 076 093 (81.68)	Neg	110	10
	SAHL77	KA	9.5	68 259 940	55 012 209 (80.59)	197	Neg	0
	LH115	AK	x	71 763 092	58 040 957 (80.88)	16	Neg	0
	LH117	AK	x	60 115 656	40 888 415 (68.02)	120	Neg	0
	LH125	AK	x	31 473 028	9 863 260 (31.34)	197	Neg	0
	SAHL68	AK	7.2	79 821 934	66 027 312 (82.72)	16	Neg	0
	CASKI	-	x	63 537 748	46 601 983 (73.35)	16	16	89461
	WATER	-	x	27 143 144	3 258 274 (12.00)	-	Neg	0
Run 3	LH116	AK	9.2	421 808 586	275 615 220 (65.34)	Neg	110	89
	SAHL77	KA	9.5	273 604 124	175 030 453 (63.97)	197	Neg	0
Run 4	LH130	AK	x	949 531 360	850 043 708 (89.52)	124, 197	Neg	0

Total reads, human reads and Human papillomavirus reads detected in all specimens included in the study. HPV: Human papillomavirus; AK: actinic keratosis; BD: Bowen´s disease; KA: keratoacanthoma; SCC: squamous cell carcinoma; RIN: RNA integrity score; x: no RIN score calculated; Neg: negative.

*HPV types found by DNA sequencing in the skin lesions were retrieved from previous paper [20] and in CaSki cell line from previous paper [18].

When the skin lesions were sequenced with multiplexing for 8 samples, only 2 specimens (both actinic keratosis) were positive for HPV RNA transcripts, one positive for HPV 16 (53 reads) and one positive for HPV 110 (10 reads) (Table 1). The HPV 16 RNA-reads detected corresponded to the spliced transcript E1^E4 (splice junction sequences were included in these reads) and the ORFs E1 and E2/E4. The HPV 110 RNA-reads mapped to the ORFs E7, E2/E4 and to the spliced transcript E1^E4. Whole genome sequencing of these tumors detected DNA from HPV 124 and HPV 197 for the first sample (HPV 16 RNA-positive) and no HPV DNA for the second one (HPV 110 RNA-positive) [20].

In order to investigate if increased sequencing depth would detect more HPV transcripts, we increased the depth 4 times by multiplexing only two lesions (the two with the highest RIN score for each of the two hospitals) (Table 1). In addition, these two lesions were particularly interesting as one was the HPV 110 RNA-positive actinic keratosis (LH116) and the other was the HPV 197 DNA-positive keratoachantoma (Sahl77). When the HPV 110 RNA-positive specimen was sequenced at a 4 times greater depth, 89 RNA reads from HPV 110 were detected, with reads that mapped to the described spliced transcript and ORFs, as well as to the L2 and E6 regions. The HPV 197 DNA-positive specimen was still negative for HPV.

HPV 16 RNA-reads were detected only in one actinic keratosis lesion (specimen LH130). No other sample nor water controls generated any HPV 16 reads. As HPV 16 is a mucosal high risk type for cervical cancer it would be interesting if an HPV 16 transcript was detected in a pre-malignant skin lesion, suggesting an active HPV 16-infection. We considered whether the HPV 16 reads found in LH130 might have resulted from contamination from the HPV 16-positive control (CaSki cell line) used in the experiment, most likely due to a phenomenon known as “index hopping”, where similar indexed adapters are mis-read during multiplexed sequencing. LH130 index was AR013 (AGTCAA) and the CaSki index was AR015 (ATGTCA). These indices only differ in an extra T at the second position in AR015 and an extra A in the last position in AR013.

Comparing sequences of HPV 16 reads from LH130 and CaSki positive controls, one mutation was detected (substitution C3470T) between specimens. While LH130 showed 87% reads (13/15) with a thymine at position 3470, CaSki controls presented 100% cytosine (21508/21610 and 13118/13143, for both CaSki positive controls).

To validate this positive HPV 16 results we sequenced this specimen at 8 times greater depth (2x75 cycles) without the CaSki positive control in the same run. No HPV reads were detected for this sample at a deeper sequencing.

The DNA and RNA detection of HPV 16 in the different skin lesions do not coincide as 2 skin lesions were positive for HPV 16 DNA, but negative for HPV 16 RNA (Table 1). An explanation for the DNA-positivity could be HPV deposition from a mucosal to the cutaneous site without a viral infection in the skin.

## Discussion

Cutaneous HPVs are commonly detected both on healthy skin [34, 35] and in skin diseases [4, 19]. However, the cutaneous HPVs are only rarely present at more than 1 viral copy per cell [15, 22–24] and HPV transcription has not been detected in skin [26, 27]. We detected and confirmed HPV RNA in 1/12 skin lesions, with HPV 110 being found in one actinic keratosis lesion. The HPV 110 reads mapped to the E1^E4 spliced transcript, which is the most abundant transcript during an HPV infection, in both low grade and high grade lesions [36–38].

In 11/12 skin lesions, we did not find any HPV expression. Besides true negativity for HPV expression, possible explanations for not finding HPVs might include: specimen inadequacy and detection method still not sensitive enough [39]. Specimen adequacy was confirmed by detection of human reference genes (ACTB, GAPDH). Positive control cells (CaSki cell line) were included in the study to confirm possible detection of HPV 16 transcription. The sensitivity of mRNA detection was assessed and increased by two approaches. First, we subjected total RNA to ribosomal RNA depletion. We didn’t opt for the standard procedure of enriching polyadenylated (poly(A)) RNA transcripts, as this method might eliminate non-poly(A) RNA sequences as well as it might not capture partially degraded mRNAs [40]. Second, we pooled less samples (sequencing run 3 contained only 2 samples) in order to achieve more throughput per sample. The total number of reads per sample increased to 270 million and 425 million reads, but no different result was obtained, hence, it is likely that the depth used in the study should have been enough to detect HPV transcription if present.

We choose to align our sequencing reads against all HPV protein sequences present in the PaVE database, not to miss a large number of recently reported HPV types, which are not established yet, and to see if we were able to detect divergent putative novel HPVs. We did not find any novel HPV type, nor did we detect any of the HPV types previously detected by DNA sequencing in the same specimens, which may suggest that these types are only present as environmental deposition rather than active infections. DNA from HPV 197 (putative type SE46 in previous papers [20, 41]) was detected in 8/12 samples, however, RNA from HPV 197 was not detected in any of the 12 samples. The absence of HPV 197 transcription in the skin lesions may suggest that HPV 197 is not involved in NMSC. The absence of DNA from the same HPV-types detected by RNA (HPV 110) may be explained by the higher abundance of HPV transcripts compared to HPV genomes in active infections.

In conclusion, RNA sequencing can detect presence of HPV transcription. While HPV is actively transcribed in cervical cancers, HPV transcription in skin lesions seems to be a rare event. Using this strategy of RNA-seq in further studies on the non-human transcriptome in NMSCs and precursor lesions may give more insight into the role of viruses in these tumors. In addition, all tumors with increased incidence among immunosuppressed patients may be analyzed in the same manner in order to investigate putative viral etiologies in those cancers.

## Supporting information

S1 Table RPKM (reads per kilobase per million) in the skin lesions and controls. Normalization of reads (RPKM units) for ACTB (actin beta) and GAPDH (Glyceraldehyde-3-Phosphate Dehydrogenase) protein coding genes for skin specimens.
(DOCX)

S1 Fig Coverage of the gene ACTB exons and introns for the HPV RNA positive samples LH116 and LH130. The height of the grey bars corresponds to the number of reads at each nucleotide position. The exons of the genes ACTB is visualized at the bottom as blue boxes and the introns as blue lines. The high coverage of most of the exons and lack of coverage of intron regions indicates that the sequences originate from RNA with no or very little DNA contamination.
(TIF)

S2 Fig Coverage of the gene GAPDH exons and introns for the HPV RNA positive samples LH116 and LH130. The height of the grey bars corresponds to the number of reads at each nucleotide position. The exons of the gene GAPDH is visualized at the bottom as blue boxes and the introns as blue lines. The high coverage of most of the exons and lack of coverage of intron regions indicates that the sequences originate from RNA with no or very little DNA contamination.
(TIF)
