# Supplementary material for: HPV transcription in skin tumors
Source: PLoS One. 2019 May 31;14(5):e0217942. doi: 10.1371/journal.pone.0217942 (PMC6544312; doi:10.1371/journal.pone.0217942)
Supplement: S1 Table — Normalization of reads (RPKM units) for ACTB (actin beta) and GAPDH (Glyceraldehyde-3-Phosphate Dehydrogenase) protein coding genes for skin specimens. (DOCX) [file pone.0217942.s001.docx]

| **Run** | **Specimen** | **RPKM** | |
| --- | --- | --- | --- |
|  |  | **ACTB** | **GAPDH** |
| 1 | LH129 | 14.85 | 35.96 |
|  | LH130 | 17.56 | 33.53 |
|  | LH137 | 11.77 | 46.78 |
|  | Sahl64 | 12.99 | 32.88 |
|  | Sahl73 | 10.21 | 40.17 |
|  | Sahl83 | 19.72 | 41.20 |
|  | CaSki | 74.84 | 311.82 |
|  | Water | 0.01 | 0.00 |
| 2 | LH116 | 12.22 | 97.81 |
|  | Sahl77 | 31.96 | 131.49 |
|  | LH115 | 14.84 | 69.25 |
|  | LH117 | 6.56 | 21.73 |
|  | LH125 | 2.20 | 3.00 |
|  | Sahl68 | 18.48 | 41.62 |
|  | CaSki | 110.53 | 323.37 |
|  | Water | 0.00 | 0.02 |
| 3 | LH116 | 8.29 | 42.90 |
|  | Sahl77 | 23.57 | 62.37 |
| 4 | LH130 | 20.84 | 44.70 |
