# Supplementary figures and images for: HPV transcription in skin tumors
Source: PLoS One. 2019 May 31;14(5):e0217942. doi: 10.1371/journal.pone.0217942 (PMC6544312; doi:10.1371/journal.pone.0217942)

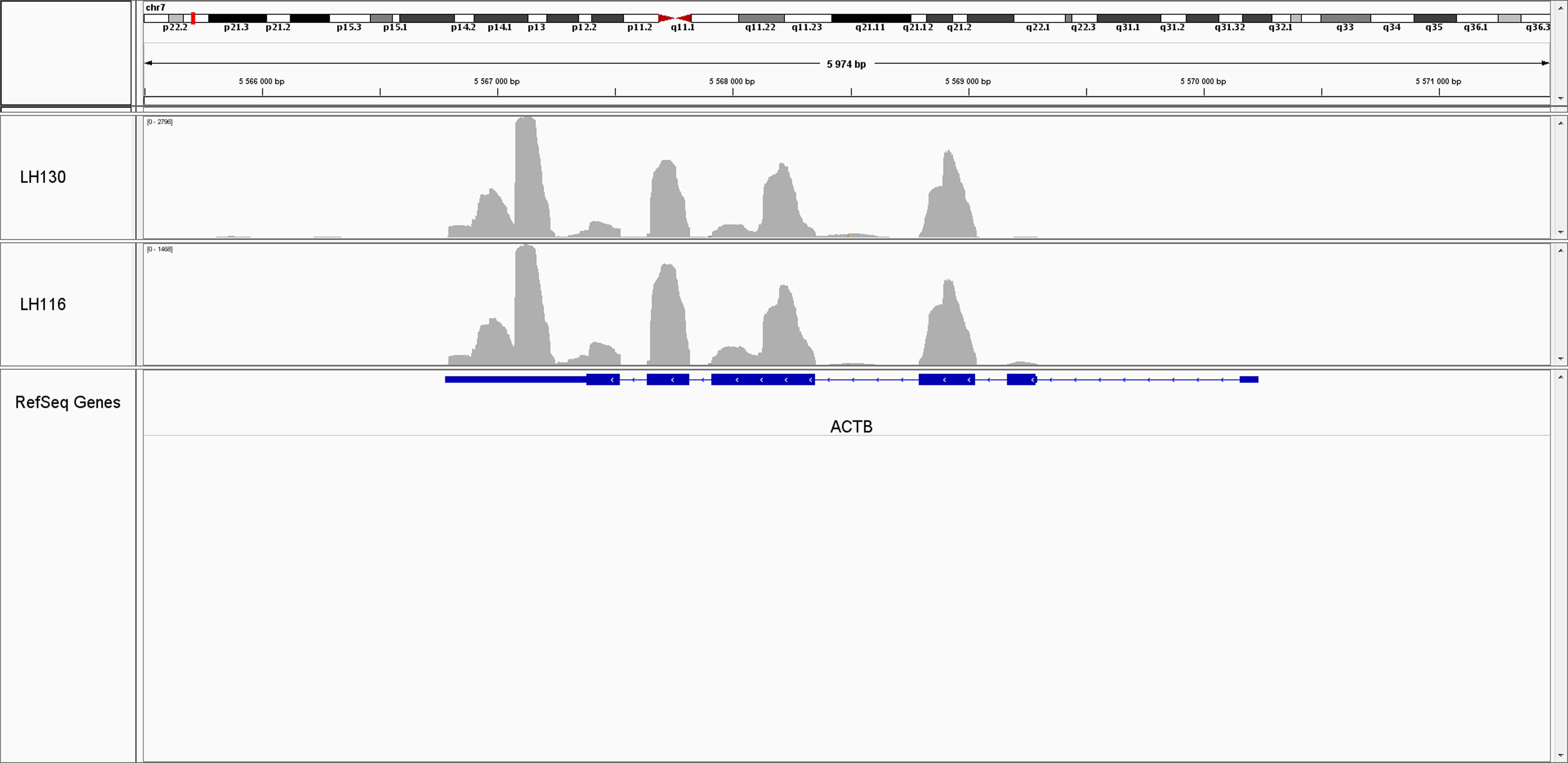

Supplement: S1 Fig — The height of the grey bars corresponds to the number of reads at each nucleotide position. The exons of the genes ACTB is visualized at the bottom as blue boxes and the introns as blue lines. The high coverage of most of the exons and lack of coverage of intron regions indicates that the sequences originate from RNA with no or very little DNA contamination. (TIF) [file pone.0217942.s002.tif]

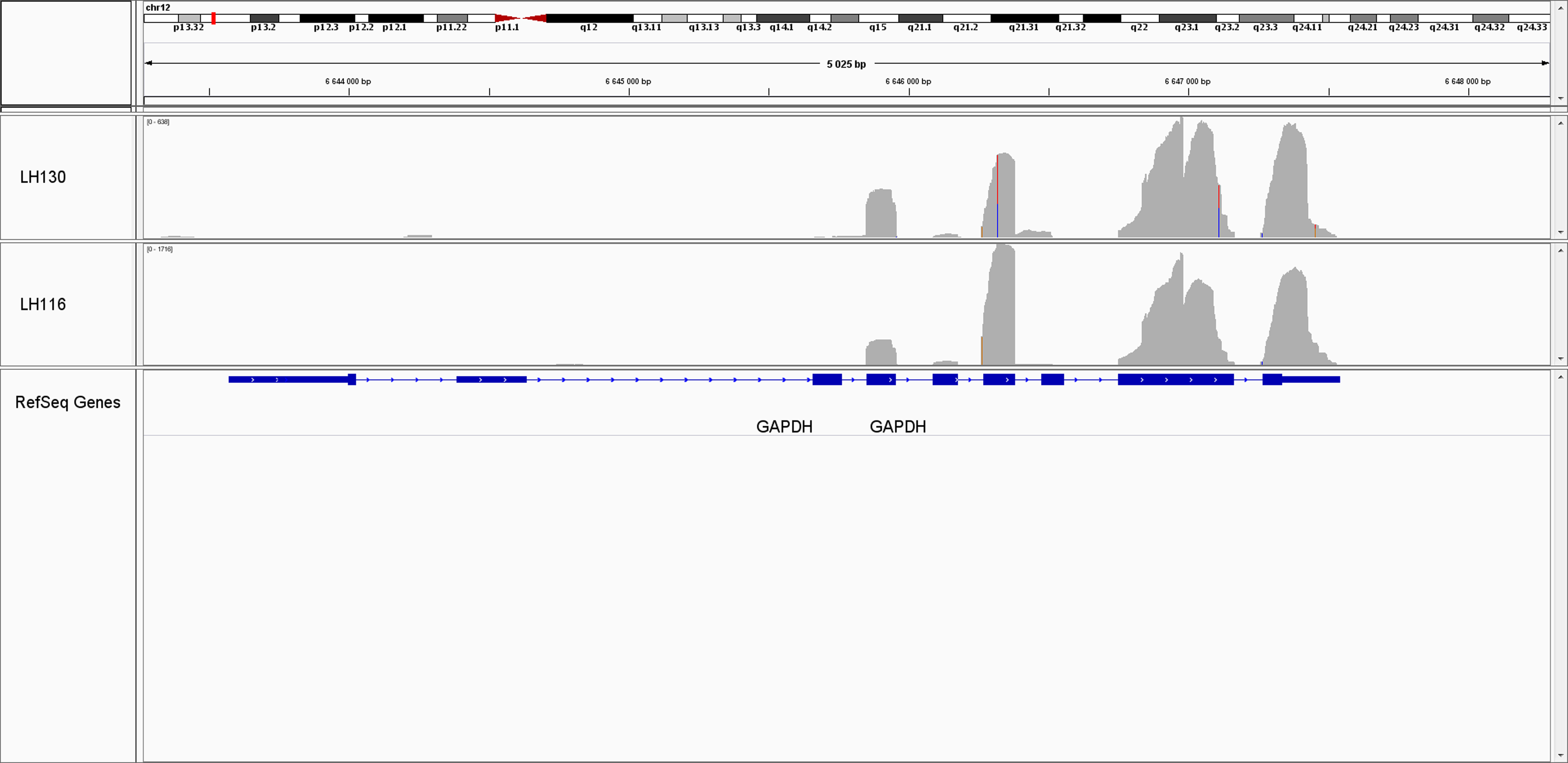

Supplement: S2 Fig — The height of the grey bars corresponds to the number of reads at each nucleotide position. The exons of the gene GAPDH is visualized at the bottom as blue boxes and the introns as blue lines. The high coverage of most of the exons and lack of coverage of intron regions indicates that the sequences originate from RNA with no or very little DNA contamination. (TIF) [file pone.0217942.s003.tif]
